# Imaging Molecular Targets and Metabolic Pathways in Breast Cancer for Improved Clinical Management: Current Practice and Future Perspectives

**DOI:** 10.3390/ijms25031575

**Published:** 2024-01-26

**Authors:** Honest Ndlovu, Ismaheel O. Lawal, Kgomotso M. G. Mokoala, Mike M. Sathekge

**Affiliations:** 1 Nuclear Medicine Research Infrastructure (NuMeRI), Steve Biko Academic Hospital, Pretoria 0001, South Africa; ndlovuhonest@gmail.com (H.N.); kgomotso.mokoala@up.ac.za (K.M.G.M.); 2 Department of Nuclear Medicine, University of Pretoria & Steve Biko Academic Hospital, Private Bag X169, Pretoria 0001, South Africa; ismaheel.opeyemi.lawal@emory.edu; 3 Department of Radiology and Imaging Sciences, Emory University, Atlanta, GA 30322, USA

**Keywords:** breast cancer, tumor heterogeneity, immunohistochemistry, receptor expression, standard imaging, tumor microenvironment, PARP imaging

## Abstract

Breast cancer is the most frequently diagnosed cancer and leading cause of cancer-related deaths worldwide. Timely decision-making that enables implementation of the most appropriate therapy or therapies is essential for achieving the best clinical outcomes in breast cancer. While clinicopathologic characteristics and immunohistochemistry have traditionally been used in decision-making, these clinical and laboratory parameters may be difficult to ascertain or be equivocal due to tumor heterogeneity. Tumor heterogeneity is described as a phenomenon characterized by spatial or temporal phenotypic variations in tumor characteristics. Spatial variations occur within tumor lesions or between lesions at a single time point while temporal variations are seen as tumor lesions evolve with time. Due to limitations associated with immunohistochemistry (which requires invasive biopsies), whole-body molecular imaging tools such as standard-of-care [^18^F]FDG and [^18^F]FES PET/CT are indispensable in addressing this conundrum. Despite their proven utility, these standard-of-care imaging methods are often unable to image a myriad of other molecular pathways associated with breast cancer. This has stimulated interest in the development of novel radiopharmaceuticals targeting other molecular pathways and processes. In this review, we discuss validated and potential roles of these standard-of-care and novel molecular approaches. These approaches’ relationships with patient clinicopathologic and immunohistochemical characteristics as well as their influence on patient management will be discussed in greater detail. This paper will also introduce and discuss the potential utility of novel PARP inhibitor-based radiopharmaceuticals as non-invasive biomarkers of PARP expression/upregulation.

## 1. Introduction

Worldwide, breast cancer has a significant contribution to cancer-related morbidity and mortality. It accounts for 30% of female-related cancers and has a mortality-to-incidence ratio of 15% [1,2]. Patient outcomes in breast cancer heavily depend on prompt decision-making that enables the implementation of appropriate treatment(s). A thorough evaluation using histological, immunohistochemical and staging approaches informs decision-making [3]. These methods seek to determine the most efficient treatments, forecast the expected path of cancer—regardless of the usage of therapies—and forecast the effectiveness of therapies [4]. Histological evaluation of tumor type and grade has traditionally served as the foundation of classification schemes for breast cancer. While growth pattern determines the tumor type, invasive ductal and lobular carcinomas account for 85% and 15% of all invasive breast carcinomas, respectively. Histological grade, which is related to the ki-67 index, determines the tumors’ proliferative index and degree of differentiation. These are classified as G1 (well-differentiated), G2 (moderately differentiated) and G3 (poorly differentiated) [5,6]. Using immunohistochemical analysis, which routinely evaluates the expression of the endocrine receptors (estrogen receptor (ER) and progesterone receptor (PR) and human epidermal growth factor-2 (HER2), breast cancer is further classified into five molecular subtypes [7,8]. Approximately 70% of breast cancer cases are ER-positive breast cancer while the remaining 30% is evenly split between triple-negative (TNBC) and HER2-enhanced breast carcinomas [9,10].

Important prognostic information relates to these immunohistochemical or molecular subtypes. ER-positive breast cancer has a favorable prognosis while HER2+ (if untreated) and TNBC have a worse prognosis [11]. On the contrary, despite invasive lobular carcinomas having the favorable prognostic characteristics of ER+ and HER2-, they are associated with a poor prognosis compared to invasive ductal carcinoma [6]. This has been explained by the greater propensity of invasive lobular carcinoma to have several genomic alterations which include phosphatidylinositol 3-kinases (PI3K) pathways and mutations in *HER2*, *HER3* and *estrogen receptor 1α*(*ESR1*) genes, amongst other factors [12]. Due to increased prevalence of these genetic characteristics, invasive lobular cancer exhibits enhanced resistance to endocrine therapies [12].

**Figure 1:**
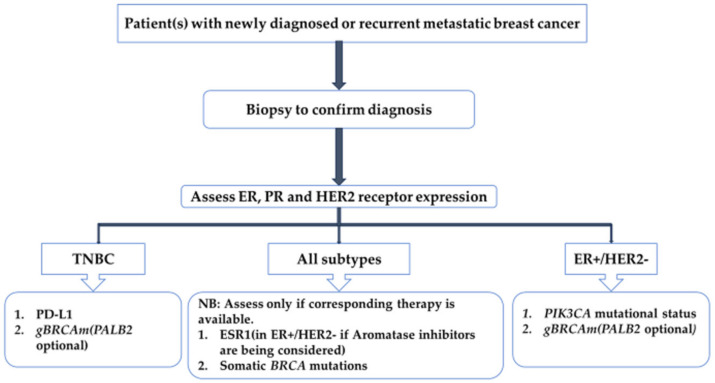
Pathological work-up in metastatic breast cancer. ER, estrogen receptor; PR, progesterone receptor; HER2, human epidermal growth factor receptor 2; PD-L1, programmed death-ligand 1; *gBRCAm*, germline *BRCA1/2* mutation; PIK3CA, phosphatidylinositol-4,5-bisphosphate 3-kinase catalytic subunit alpha; TNBC, triple-negative breast cancer; ESR1, estrogen receptor 1α.

Immunohistochemistry also plays a major role in selecting appropriate therapy in the neoadjuvant or adjuvant settings. Classical examples are the use of the US Food and Drug Administration (FDA)-approved endocrine/hormonal therapies targeting ER or HER2 receptors or their downward pathways which have demonstrated favorable survival outcomes [13,14]. Depending on the availability of relevant therapies, other novel therapeutic relevant biomarkers may be incorporated into a patient’s work-up. These biomarkers include and are not limited to germline *breast cancer gene 1/2*(*BRCA1/2)* mutation (*gBRCAm*) in HER2-negative, programmed death-1 ligand (PD-L1) status in TNBC and phosphatidyl inositol-4,5-bisphosphonate 3-kinase catalytic sub-unit alpha (PIKA3CA) in ER/PR+ HER2-negative cancers [3]. The relevant clinically approved therapies include immune checkpoint inhibitors pembrolizumab used in combination with chemotherapy for the treatment of high-risk TNBC and cyclin-dependent kinase (CDK) 4 and 6 inhibitor abemaciclib approved as adjuvant treatment of patients with high-risk hormone receptor (HR)-positive disease [15,16]. Recently, poly adenosine diphosphate ribosyl (PARP) inhibitors have been incorporated and have shown significant outcome benefits in patients with pathogenic/likely pathogenic *BRCA1*/2 variants/mutations and high-risk HER2-negative breast cancer [17,18,19]. Figure 1 provides a comprehensive guide to the laboratory work-up in metastatic breast cancer.

The gold standard for determining receptor expression and molecular markers in breast cancer is immunohistochemistry [20]. Despite similar immunohistochemical features, responses are variable across patients, with some patients showing little to no benefit from these therapies. Tumor heterogeneity is the most likely explanation for the differences in behavior of these tumors with similar immunohistochemical characteristics [21]. This occurs when tumors, despite sharing many characteristics, have distinct morphological and phenotypic profiles which affect their metabolism, proliferation and metastatic potential [21,22]. Mutational evolution of the cancer, wherein the cancer behaves as an evolving ecosystem under the selective pressures applied by both the host immune response and anticancer therapeutics, has been proposed as the likely cause for tumor heterogeneity [23]. Mutation evolution occurs in either parallel or linear progression. In linear progression, a small and advanced clone of the primary tumor that has not been detected due to the intrinsic heterogeneous nature of the primary tumor spreads to a distant site. In parallel progression, the main component detected metastasizes and mutates at a distant metastatic site. This tumor heterogeneity may be within the primary tumor due to its polyclonal nature. Depending on the type and the number of cellular clones(s) of primary lesion that metastasize, the metastatic lesion may either be polyclonal or monoclonal [22,24]. 

Heterogeneity has been demonstrated in hormone and HER2 receptor expression [25,26]. Also, metastatic breast cancer histological subtype differs from the original tumor classification in approximately 17% of recurrent MBC patients when they switch from lobular to ductal and vice versa [27]. To note is that other receptors have demonstrated a role in the functionality of the ER by conferring resistance to therapy targeted towards ER and downstream pathways. For example, when almost all patients with ER+ breast cancer develop resistance to ER-directed agents in the metastatic setting, it is attributed to mutations of HER2 in addition to the ER mutations [28].

In this regard, pre-treatment histological and immunohistochemical assessment should ideally be supplemented by dynamic biopsies of primary recurrent or metastatic sites. This is precluded by the invasiveness, patient inconvenience and the inherent risk of missing some cellular components due to tumor heterogeneity during histological staining. Therefore, non-invasive in vivo whole-body assessment tools become important in selecting patients who are more likely to benefit from these expensive therapies which are not devoid of serious side effects. 

Morphological imaging techniques, which include ultrasound (USS), X-ray, computed tomography (CT), magnetic resonance imaging (MRI) and molecular imaging techniques, that include a bone scan and [^18^F]fluoro-2-deoxyglucose positron emission tomography/computed tomography([^18^F]FDG PET/CT), have been used in the evaluation of metastatic breast cancer. These imaging techniques have found major roles in staging, response assessment, restaging, recurrence assessment and as predictors of outcomes in breast cancer. These have relative advantages and disadvantages as has been comprehensively discussed in the literature [29,30]. 

In the era of precision/personalized medicine, imaging methods tailored to a specific patient or cancer characteristic have become a vital need. Molecular imaging capable of visualization, characterization and measurement of various biological processes at both molecular and cellular levels has been instrumental in this regard [31]. The current standard-of-care [^18^F]FDG PET/CT, which targets glucose metabolism in tumors, and the recently approved [^18^F]FES PET/CT, as a biomarker of estrogen receptor expression, have been at the forefront of breast cancer management. Despite their proven utility, these standard-of-care imaging methods are often unable to image a myriad of other molecular pathways associated with breast cancer. This has stimulated interest in the development of novel radiopharmaceuticals targeting other molecular pathways and processes. In this review, we discuss the validated and potential roles of these standard-of-care and novel molecular approaches. Their relationship with patient clinicopathologic and immunohistochemical characteristics, as well as their influence on patient management will be discussed. The paper will also introduce and discuss the potential utility of novel PARP inhibitor-based radiopharmaceuticals as non-invasive biomarkers of PARP expression/upregulation.

## 2. Current Standard-of-Care PET Imaging Techniques in Breast Cancer Management

### 2.1. [^18^F]FDG PET/CT

The role of [^18^F]FDG PET/CT in breast cancer has been validated in pre-treatment staging, response assessment, restaging (known or suspected recurrence) and prognostication/prediction of response in many tumors [3,32]. The 2022 European Society for Medical Oncology (ESMO) and the National Comprehensive Cancer Network (NCCN) guidelines recommend CT and bone scan/[^18^F]NaF as the standard for staging [3,32]. The ESMO guidelines also recommend that [^18^F]FDG PET/CT, if available, may be used in place of these [3]. Both guidelines acknowledge the usefulness of [^18^F]FDG PET/CT in detecting unsuspected regional lymph nodes and/or distant metastases as an added advantage compared to CT and bone scans. The guidelines recommend [^18^F]FDG PET/CT when findings noted on CT and bone scan are equivocal [3,32]. The rationale of tumor imaging with [^18^F]FDG PET/CT is based on the premise that in aggressive/invasive cancer such as breast cancer, glucose metabolism as a primary source of energy and metabolic substrates is upregulated [33]. Thus, increased glucose utilization can be traced with [^18^F]FDG a radiolabeled glucose analog. In this tracer, the hydroxyl- group in position 2 of glucose is replaced by [^18^F]F [34]. After the uptake of [^18^F]FDG by the tumor cell, it is phosphorylated to [^18^F]FDG-6-phosphate as part of the first step of glycolysis. Further phosphorylation does not take place due to the absence of the hydroxyl group in position 2. This and other factors such as the downregulation of glucose-6-phosphorylase and upregulation of hexokinase result in the accumulation of glucose/[^18^F]FDG in these tumor cells [33,34,35]. The uptake of [^18^F]FDG imaged with PET/CT can be analyzed with both visual and semi-quantitative methods (see Table 1). The CT component may either serve as a low-dose CT for attenuation correction and anatomical co-localization or a high-dose diagnostic CT. This increases the spatial and anatomic resolution of the study. It also provides an evaluation of the morphological characteristics of the tumor. These PET and CT characteristics are invaluable in the objective response assessment and follow-up in these patients [36].

#### 2.1.1. [^18^F]FDG Uptake Patterns Based on the Histological and Immunohistochemical Subtype of Breast Cancer

The two main breast cancer histological subtypes demonstrate different [^18^F]FDG uptake patterns which are important in recommending, reporting and interpreting [^18^F]FDG PET/CT scans [37]. This relates to both primary and metastatic lesions. Ductal carcinomas have higher uptake or SUV values compared to lobular carcinomas [37]. In the same vein, the predominantly skeletal metastases seen in invasive lobular cancer demonstrate low-grade to absent [^18^F]FDG uptake. In contrast, invasive ductal carcinomas with predominantly osteolytic skeletal metastases are associated with high [^18^F]FDG uptake and SUV values [38]. Other clinicopathological factors that are associated with high [^18^F]FDG uptake or SUVmax values include Ki-67 index/higher nuclear grade, larger tumor size, positive nuclear grade and higher tumor node metastasis (TNM) stage [37,39]. Kitajima et al. demonstrated a correlation of [^18^F]FDG uptake with the immunohistochemical subtype of breast cancer [39]. In their univariate analysis, ER and PR negativity, HER2 positivity and high Ki-67 index were associated with high SUV max. In multivariate analysis, Kitajima et al. showed higher SUVmax values in TNBC. This was followed by HER2-enhanced and luminal B while luminal A had the lowest values [39]. Sasada and colleagues reported similar findings. They echo the same sentiments that a semi-quantitative retention index (RI) calculated based on SUVmax at two time points could be used to differentiate between the immunohistochemical subtypes of breast cancer [40]. Sasada and colleagues showed that TNBC is associated with the highest RI followed by HER2 positive, luminal B and lastly luminal A breast cancer [40]. Cementing the above findings, Yoon et al., reported that [^18^F]FDG uptake in patients with untreated invasive ductal carcinoma is inversely correlated to the hormone receptor status [41]. Greohuex and colleagues reported a relationship between *p53*-mutated tumors with high SUVmax and poor prognostic markers such as high tumor grade, hormone receptor negativity and triple negativity [42]. They did not show any association with HER2 receptor status while hypothesizing that the HER2 receptor has less impact on the glycolytic pathway which they however confirmed later on in their follow-up study [42,43]. Contrary to this, regardless of HER2 or endocrine receptor expression, tumors expressing epidermal growth factor receptor (EGFR) also known as HER1 are associated with high SUVmax values [44]. The influence of these clinicopathologic and molecular elements on [^18^F]FDG uptake emphasizes the need for alternative imaging probes where applicable.

#### 2.1.2. The Role of [^18^F]FDG PET/CT in Breast Cancer

##### Staging

In the era of precision medicine, the individualization of investigations/therapy by considering clinicopathological and immunohistochemical factors is of utmost importance in treatment decision-making [45]. The NCCN and ESMO guidelines acknowledge that [^18^F]FDG PET/CT is useful in equivocal cases as well as in identifying unsuspected regional or distant metastases. These guidelines advise against using it routinely in clinical stages I, II and operable III(T3N1) [3,32]. This is due to the high incidence of false positives, false negatives and low sensitivity of micrometastases in axillary lymph node metastases [32]. The guidelines are not one-size-fits-all; they should be interpreted in the context of a risk-stratified/pre-test probability approach by considering other patient factors. This may reduce the incidence of patients undergoing suboptimal or futile invasive therapies which may result in less-than-ideal results [45]. Although [^18^F]FDG PET/CT is not advised in stage II breast cancers, unsuspected metastases have been detected on [^18^F]FDG PET/CT. Ulaner et al., demonstrated the effectiveness of [^18^F]FDG PET/CT in early-stage breast cancer. They assert that when [^18^F]FDG PET/CT is used in newly diagnosed ER-positive/HER2-negative, breast cancers revealed 4% of unanticipated distant metastases in newly diagnosed in stage I while 14% and 26% were recorded in unanticipated distant metastases in newly diagnosed initial stages II and IIB, respectively [46]. Similar results with other immunohistochemical subtypes have been reported, showing 15% and 11% of unsuspected distant metastases in TNBC and other mixed receptor phenotypes. Overall, these studies show that [^18^F]FDG PET/CT has the potential to upstage 15% of stage IIB breast cancer across all receptor phenotypes. This has also been reported in male breast cancer [4,47,48,49]. [^18^F]FDG PET/CT has other advantages over bone scans and morphological imaging modalities. These include mammogram, breast ultrasound, abdominal ultrasound and CT, X-rays, and CT of the chest. Cochet et al. evaluated its impact on management and prognosis in patients with newly diagnosed breast cancer (>2 cm or >T1). [^18^F]FDG PET/CT upstaged 21% and downstaged 16% of patients based on staging using other modalities [50]. The use of other modalities was seen to be time-consuming whereas [^18^F]FDG PET/CT was an efficient one-stop shop. The authors also assessed the impact of [^18^F]FDG PET/CT on patient management by comparing pre- and post-PET/CT management plans. [^18^F]FDG PET/CT demonstrated high impact on a substantial number of patients necessitating a change in the treatment plan or the method of treatment delivery [50]. Cochet et al.’s study also found high SUVmax values in ductal, HER2-enhanced and TNBC. In the same study, one lesion was equivocal on bone scan and negative on [^18^F] FDG PET, making it indeterminate. Histological evaluation of the lesion, if performed, could have revealed lobular carcinoma which is associated with poor prognosis and variable metabolic activity [50]. The association of lobular carcinomas with ER positivity means that the patient may benefit from ER-targeted imaging and therapy [51].

Magnetic resonance imaging (MRI) produces images with good contrast morphological information and excellent spatial resolution [52]. The advances in magnetic contrast agents have significantly increased the sensitivity of MRI. These contrast agents are either paramagnetic ion complexes or superparamagnetic particles [53]. They contain lanthanides like gadolinium or transitional metals such as manganese. These act by shortening T1 or T2 relaxation times which results in increased intensity of T1-weighted images or T2-weighted images, respectively [54]. Amongst these magnetic contrast agents, gadolinium (III) based contrast agents (GBCAs) were the first to gain widespread use. These GBCAs have however been associated with increased in vivo residence times and potentially fatal systemic fibrosis which has led to the development of superparamagnetic iron oxide nanoparticles (SPIONs) as potential alternatives [54,55]. SPIONs have distinct properties that make them feasible alternatives such as simple metabolism, biocompatibility, high saturation magnetic moments, which may be adjusted by particle size and composition, and easy surface functionalization. The fact that SPIONs are size-restricted by the superparamagnetic regime has been a deterrent to breaking into the market despite their approval [55,56]. Magnetic nanostructure (MNS), particularly nanodiscs and nanowires, are potential alternatives to SPIONs because of their greater magnetic moments that are not constrained by the superparamagnetic domain [52,56]. MNSs are also a viable system for theranostics capable of use as contrast agents and generating localized heat inside the body using mechanisms such an external alternating current magnetic field [52].

With the use of these magnetic contrast agents such as GBCA, MRI can map blood flow, especially in soft tissue such as neural tissue. It plays a pivotal role in treatment planning by evaluating the extent of primary or metastatic disease invasion into vital structures such as the brain and spinal cord. This is important because, in these areas, [^18^F]FDG PET/CT has a low diagnostic yield [57]. [^18^F]FDG imaged with PET/CT and the newer PET/MRI is seen to outperform CT or MRI in detecting nodal involvement on both patient- and lesion-based analysis with high sensitivity. In a study by Morawitz et al., PET/MRI detected 193 lesions in 75 patients (41.2%) while MRI detected 123 lesions in 56 patients (30.8%). CT on the other hand detected 104 lesions in 50 patients [58]. A comparison of PET/MRI and PET/CT is discussed in the recurrence assessment section.

##### Response Assessment

The response assessment criteria in solid tumors (RECIST 1.1) has been a cornerstone for response assessment in solid tumors by quantifying size changes in measurable lesions on CT [59]. The exclusion of non-measurable lesions (e.g., leptomeningeal disease, ascites, pleural or pericardial effusions, inflammatory breast disease, lymphangitic involvement of skin or lung, skeletal, and some lesions below a crucial diameter, such as fewer than 10 mm metastases) that are associated with poor prognosis is an unignorable pitfall of RECIST 1.1 [59]. [^18^F]FDG PET plays a pivotal role in the assessment of lesions based on uptake using the SULpeak regardless of size changes as part of the PET response criteria in solid tumors 1.0 (PERCIST) [60]. RECIST 1.1 and PERCIST 1.0 have been compared by Riedl et al. All patients deemed responders on RECIST 1.1 were responders on PERCIST 1.0. However, 40% that were non-responders on RECIST 1.1 were responders on PERCIST 1.0 [61]. The superiority of PERCIST 1.0 was seen in the follow-up of these patients [61]. Although responses according to RECIST and PERCIST both correlated with progression-free survival (PFS), PERCIST 1.0 showed significantly higher predictive accuracy (concordance index for PFS: 0.70 vs. 0.60). One-year PFS for responders vs. non-responders by RECIST 1.1 was 59% vs. 27% compared to 63% vs. 0% by PERCIST. Four-year disease-specific survival (DSS) of responders and non-responders by RECIST was 50% and 38%, respectively, compared to 58% vs. 18% for PERCIST 5. Response on PET/CT was a significantly better predictor for DSS than disease control on CE-CT [61].

Early response assessment allows unbeneficial therapies with untoward side effects to be halted early. Early response assessment using PERCIST 1.0 has also demonstrated superiority over RECIST 1.1. In a longitudinal study by Vogsen et al., disease progression was seen first on [^18^F]FDG PET/CT in 43 patients (49.4%) and on CE-CT in 1 patient (1.15%) out of 87 patients [62]. After excluding responders, this translated to 43 out of 55 (78.2%) and 1 out of 55 (1.82%) for [^18^F]FDG PET/CT and contrast-enhanced CT (CE-CT), respectively. The median time to detection was between 14.9 and 24.3 months while the median time from detection of progression by [^18^F]FDG PET/CT to detection by CE-CT was 5.98 months [62]. This existing evidence strengthens the superiority of PERCIST 1.0 compared to RECIST 1.1, making [^18^F]FDG PET/CT superior to CE-CT in response assessment for both. 

##### Prognostication

The process of decision-making on either curative or palliative therapies is partly governed by the patient’s prognosis and wishes. The prognosis, as determined by the clinician, can be predicted using the patient’s disease stage based on clinical examination and relevant investigations [63]. [^18^F]FDG PET/CT has found a role and is gaining traction in prognostication in breast cancer. As demonstrated by Koolen et al., [^18^F]FDG uptake represented by SUVmax is significantly higher in primary breast tumors with prognostically unfavorable characteristics [64]. Vicente et al. and Nakajo et al. echo the same sentiments, emphasizing that [^18^F]FDG PET/CT provides prognostic information in breast cancer patients pre-operatively [65,66]. They provide cut-off SUVmax values of 4.2–6.5 for primary breast cancer and 2.25 for lymph node metastases [66,67]. Other independent prognostic markers include whole-body TLG and MTV in both pre- and post-operative settings in all breast cancer immunohistochemical subtypes [68,69,70,71]. In other studies, the lymph node parameters rather than primary tumor metabolic and volumetric parameters were more significant predictors of recurrence and for evaluating prognosis [69,70].

Cochet et al. compare the prognostic role of [^18^F]FDG to that of conventional imaging methods [50]. They found that although conventional imaging showed a significant association with progression-free survival, [^18^F]FDG PET/CT provided stronger prognostic stratification. In both univariate and multivariate analysis, [^18^F]FDG PET/CT was associated with progression-free survival [50]. The role of [^18^F]FDG in predicting prognosis has also been emphasized by the association of the Ki-67 index, which is a marker of cellular proliferation and SUVmax [37,72,73,74]. An example is TNBC, which has high Ki-67 indices and high SUVmax values, being associated with poor outcomes [75].

##### Recurrence Assessment

Outcomes in recurrent breast cancer are reliant on early detection and prompt institution of the appropriate therapy. Recurrence may be suspected on clinical grounds or based on rising tumor markers such as cancer embryonic antigen (CEA), cancer antigen 15-3 (CA 15-3) and less commonly cancer antigen 19-9 (CA19-9) and cancer antigen 125 (CA125) [76]. These demonstrate variable sensitivities and specificities of 56.7% and 92%, respectively, for CEA, 36% and 82.5% for CA19-9, 25.5% and 97% for CA125 and 44.5% and 84.5% for CA15-3 [76]. Although some improvements are noted when these are combined, they have a limited role in localizing the tumor recurrence, which is important in treatment decision-making [76,77]. Therefore, imaging tools become indispensable under such circumstances. This includes conventional morphological and molecular imaging tools.

The performance of [^18^F]FDG PET/CT in recurrent breast cancer comparing it to tumor markers and conventional morphological imaging has been extensively evaluated. In patients with rising tumor markers and negative morphological imaging, [^18^F]FDG PET/CT was able to detect recurrent primary or metastatic breast cancer in 45% of cases [78]. Aukema et al. explained that [^18^F]FDG PET/CT, in addition to the lesions detected on morphological imaging, detected more lesions, changing management in 48% of patients from local curative to systemic therapy [79]. The morphological imaging methods excluded whole-body MRI which compared differently than others to [^18^F]FDG PET/CT. Schmidt et al. asserted that MRI has a better sensitivity but lesser specificity than [^18^F] FDG PET/CT: 93% vs. 91% and 86% vs. 90% [80]. The two were seen to perform differently in different circumstances. In one patient, the breast lesion which was positive on MRI and negative [^18^F]FDG PET/CT on follow-up imaging, which was considered the reference or gold standard, was confirmed to be a post-treatment change [80]. [^18^F]FDG performed better in lymph node detection both loco-regionally and extra-axillary with a diagnostic accuracy of 96% vs. 75% for MRI. On the other hand, MRI outperformed PET/CT in detecting bone, liver and lung metastases [80]. The importance of this is multi-fold. For radiation protection reasons, MRI may be preferable despite its long acquisition period while [^18^F]FDG PET/CT becomes the imaging of choice in patients with metallic implants where MRI is contraindicated. Other scenarios where [^18^F]FDG may be preferable include suspected primary disease and loco-regional disease. The complementary role of PET and MRI using the new PET/MRI scanners may be exploited [81]. The exclusion of CT in this study also results in a reduction in the patient radiation dose. However, PET/MRI scanners are not in routine use due to their limited availability [81].

#### 2.1.3. Limitations of [^18^F]FDG PET/CT Relevant to Tumor Heterogeneity

Despite its validated role and correlation to patients’ clinicopathological characteristics in breast cancer, [^18^F]FDG is not devoid of limitations. These are mainly related to breast cancer tumor heterogeneity [82]. Its indirect correlation to tumor characteristics has been mentioned. Some of these include the low SUVmax or absent metabolic activity in most ER receptor-expressing tumors such as luminal A or lobular breast cancer which may be missed on [^18^F]FDG PET/CT [82]. Therefore, the evaluation of the specific disease process using the multi-tracer approach may be of more value than imaging with a non-specific tracer like [^18^F]FDG. More useful information may be obtained from the imaging of both ER-positivity and tumor glucose metabolism than the imaging of either process without the other [83,84,85].

### 2.2. Estrogen Receptor Imaging

#### 2.2.1. Estrogen Receptor Signaling and Therapeutic Targets

**Figure 2:**
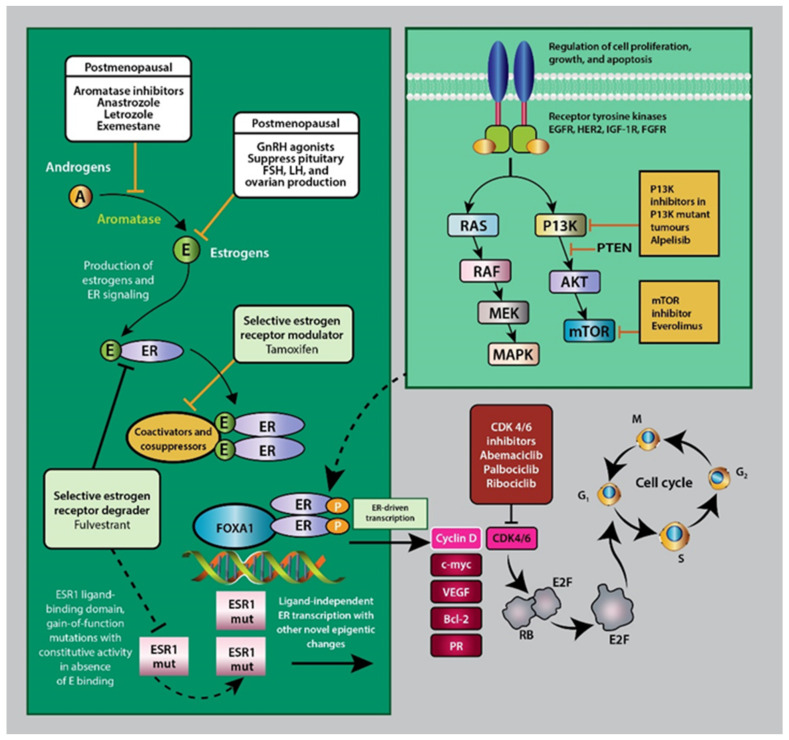
Representation of the mechanism action of estrogen and ER receptor signaling. Included are the molecular targets of various hormonal therapies targeting estrogen production, function and signaling. ER: estrogen receptor; FOXA1: forkhead protein; ESR1: estrogen receptor alpha-1; mut: mutation; EGFR: epidermal growth factor receptor; HER2: human epidermal growth factor receptor 2; IGFR1: insulin-like growth factor receptor 1; FGFR: fibroblast growth factor receptor 1, VEGF: vascular endothelial growth factor. Adapted from Harold et al. [11].

Knowledge of endocrine receptor expression is important for both treatment selection and prognosis. It has facilitated the development of prognostic staging, which has been adopted by the American Joint Committee on Cancer (AJCC) staging system in the 8th edition [86]. Estrogen and ER signaling promote tumorigenesis, proliferation and metastasis in ER-positive breast cancer. This has facilitated the development of therapies targeting estrogen production, ER signaling and mutational mechanisms (see Figure 2) [16,84].

Immunohistochemistry is the gold standard for the determination of receptor expression in breast cancer. The American Society of Clinical Oncology/College of American Pathologists (ASCO/CAP) regards breast cancer as positive if ≥1% of tumor cells demonstrate positive nuclear staining by immunohistochemistry [86,87]. Tumors expressing ER receptors in 1–9% tumor cells have been regarded as low ER-positive tumors [85] due to their limited response to ER-targeted therapies and their resemblance to TNBC, particularly if they are PR-negative [86]. The poor prognosis seen in this low ER-positive breast cancer further emphasizes the overlapping behaviors with TNBC [88]. Immunohistochemistry is not without pitfalls. There are procedural pitfalls which may deem the specimen uninterpretable due to interference or inadequate tumor sample. It is worth noting that in 20% of ER-positive breast cancer, there is discordance in ER expression between the primary tumor and metastatic lesions [89]. Furthermore, the ER’s mere presence does not imply its functionality. Therefore, if all these factors are not considered, futile and toxic therapies such as the aromatase inhibitor anastrozole, which has been associated with myocardial infarction, may be instituted [25].

From the circumstances outlined above, a whole-body assessment tool that assesses both ER expression and function becomes of value in supplementing immunohistochemistry of biopsy specimens. Various non-invasive and in vivo whole-body assessment imaging probes have been evaluated. 16-[^18^F]-fluoro-17-estradiol ([^18^F]FES) Cerianna^TM^ is an estrogen analog and the first estrogen PET imaging agent approved by the FDA for the detection of ER-positive lesions [90]. Recommended indications for [^18^F]FES PET/CT include a predictive biomarker for the efficacy of hormonal therapies, ER assessment of difficult-to-biopsy lesions and problem-solving in clinical dilemmas or inconclusive anatomical imaging. [^18^F]FES PET/CT has also been found useful in systemic staging and in guiding the evaluation of ER-targeted therapies in ER-positive breast cancer. These are discussed in the relevant sections below [90,91,92].

#### 2.2.2. Role of [^18^F]FES Breast Cancer

##### Diagnosis and Staging

[^18^F]FES PET/CT has demonstrated success in solving clinical quagmires in the diagnosis and staging of ER-positive breast cancers. In a study by Boers et al., [^18^F]FES was able to solve dilemmas in 87% of equivocal cases on standard imaging [51]. Significantly, this study included a large population of lobular cancer which are more likely to present with diagnostic dilemmas as discussed previously [51]. The propensity of fewer false negatives compared to [^18^F]FDG in ER-positive breast cancer such as in sclerotic skeletal metastases seen in invasive lobular breast cancer is associated with other downstream benefits [93]. These include reducing unwanted biopsies, reducing delays in treatment, prompt relief of symptoms and reduction in the financial burden [94,95]. This does not imply that [^18^F]FES PET/CT is not without pitfalls. False positives have been reported in benign and malignant conditions such as radiation pneumonitis and gynecological tumors [91]. False negatives are most common in liver metastases due to intense liver uptake and in patients on estrogen-targeting therapies [91].

##### Response Assessment and Prognostication

Peterson et al. believe that [^18^F]FES is effective as a measure of ER expression in newly diagnosed metastatic breast cancer. They are of the view that low uptake is correlated to lack of response to endocrine therapy and lack of ER tumor status on immunohistochemistry [96]. Chae et al. demonstrates similar results of a high negative percent agreement (100%) with a moderate positive agreement (76.6%) between [^18^F]FES and ER status on immunohistochemistry [97]. They also note that patients who had a positive [^18^F]FES had a significantly higher PR expression than those with positive ER on immunohistochemistry and negative imaging. This emphasizes the influence of PR on the ER signaling and function [97]. They further postulate that low ER uptake on [^18^F]FES may be associated with ER-positive immunohistochemistry and a lack of ER function due to ER mutations or splice variants [97]. This gives [^18^F]FES PET/CT an advantage over immunohistochemistry since it images both expression and function while the latter detects only receptor expression [98]. It should be emphasized that paradoxical metabolic flare seen with [^18^F]FDG in patients on ER-targeted therapy is not seen with [^18^F]FES PET/CT [99].

##### Recurrence Assessment

[^18^F]FES PET/CT has demonstrated value in distinguishing ER-positive malignancies from other malignancies [100]. This is particularly important in patients with a history of dual malignancies. Yang et al., are of the view that [^18^F]FES PET/CT is valuable in treatment decision-making in patients with previously treated recurrent ER-positive breast cancer and other new synchronous or metachronous malignancy developing metastatic diseases [100]. They affirmed that in 28 out of 32 patients, [^18^F]FES PET/CT played a valuable role in decision-making strategy either by changing or reassuring the physicians on the management that they decided on [100]. Their study excluded ovarian cancers which are more likely to be ER-positive. Furthermore, their study did not factor in the likelihood of ER negativity in previously ER-positive patients due to mutational evolution [100]. This could be addressed by performing both [^18^F]FES PET/CT and [^18^F]FDG PET/CT [101]. Chae et al., in their study of recurrent ER-positive breast cancer, found that 9 out of 32 patients with no uptake on [^18^F]FES demonstrated uptake on [^18^F]FDG PET/CT [101].

## 3. Promising PET Radiopharmaceuticals for Breast Cancer Imaging

Several novel radiolabeled imaging probes targeting other molecular markers or processes associated with breast cancer have shown promise in the management of breast cancer. These and their potential clinical applications are discussed in this section. Special emphasis is made on the new radiolabeled poly (ADP-ribosyl) polymerase inhibitors (PARPi) that target poly (ADP-ribosyl) polymerase (PARP) upregulation.

### 3.1. Progesterone and HER2 Receptor Expression

HER2 receptor-targeted therapies such as trastuzumab confer improved survival outcomes in patients with metastatic HER2 receptor-positive breast cancer [102]. Like ER receptor expression, HER2 receptor expression demonstrates heterogeneity. Discordancy within a primary or metastatic lesion and between lesions is also seen [103]. Most important spatial and temporal tumor heterogeneity is seen with a change in HER2 receptor expression within lesions during therapy [104]. Therefore, dynamic assessment of HER2 receptor expression in breast cancer is of paramount importance. Immunohistochemistry may not be technically feasible due to the need for invasive biopsies making whole-body imaging a feasible option. Both PET- and SPECT-based radiotracers have demonstrated the potential in improving decision-making especially if HER2 receptor expression is equivocal. Another important application is their ability to confirm HER2 receptor expression in lesions that are inaccessible for biopsy. [^89^Zr]Zr-trastuzumab is one PET tracer that has gained utility in this space [105,106,107,108]. Whole-body PR receptor expression has been imaged with [^18^F]-fluorofuranyl norprogesterone (FNNP) [109]. Unlike [^18^F]FES for ER receptor imaging, these HER2 and PR imaging agents are still under investigation.

### 3.2. Imaging the Tumor Micro-Environment (TME)

Tumor micro-environment (TME) is a topical issue in oncology. Its constituents include tumor cells, tumor stromal cells (including various types of fibroblasts), endothelial cells, immune cells and non-cellular extra-cellular matrix such as collagen [110]. The non-cancerous cells promote tumorigenesis, tumor progression and metastases [110,111]. New molecular imaging agents have targeted neo-vasculature (endothelial cells) and cancer-associated fibroblasts (CAF). These are discussed in this subsequent section.

#### 3.2.1. Cancer-Associated Fibroblasts (CAF) and Fibroblast Activated Protein (FAP)

**Figure 3:**
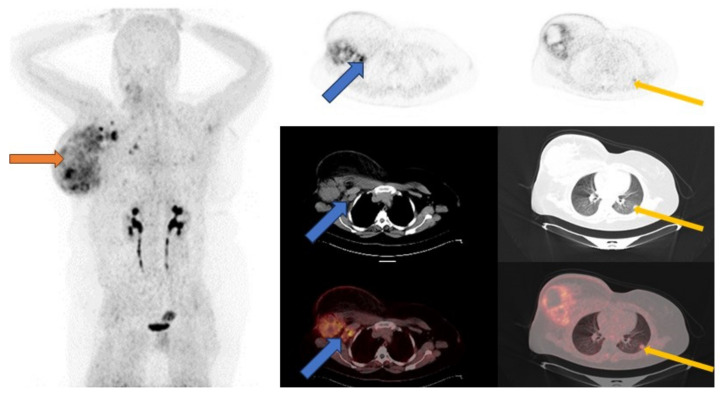
A 39-year-old female referred with locally advanced right breast cancer (TNBC). [^68^Ga]Ga-FAPi-04 PET/CT was performed. Maximum intensity projection (MIP) A: physiological urinary excretion of tracer. Non-benign uptake is also noted on the MIP. This localizes to the enlarged right breast mass (orange arrows), lymph nodes (blue arrows) and left lung (yellow arrows).

**Figure 4:**
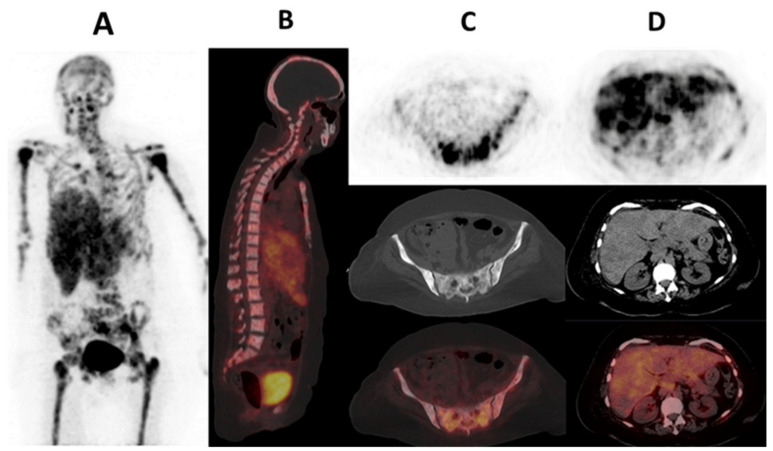
A 51-year-old woman known with metastatic invasive lobular breast cancer estrogen and progesterone receptor-positive, and HER2-negative and a Ki-67 of 45%. She had a bilateral modified radical mastectomy with disease progression despite chemotherapy. She had [^68^Ga]Ga-FAPi-04 PET/CT performed which showed diffuse skeletal metastases (**A**–**C**) in a pattern consistent with bone marrow distribution as well as liver metastases (**D**).

Cancer-associated fibroblasts (CAFs), which are part of the tumor-supporting stroma, are believed to originate from quiescent tissue resident fibroblasts, pancreatic, hepatic stellate, bone marrow-derived mesenchymal, stem, endothelial and fat cells [112]. They express a variety of receptors such as αSMA, fibroblast-specific protein 1 (FSP1, also known as S100A4), fibroblast activation protein (FAP), platelet-derived growth factor receptor-α (PDGFRα), PDGFRβ, desmin, discoidin domain-containing receptor 2 (DDR2) and vimentin [113]. These CAFs have demonstrated heterogeneity in various solid tumors including breast cancer [114,115]. Piwocka et al. and Musielak et al., using flow cytometry, immunofluorescence and reverse transcription, noted that biomarkers/receptor expression in CAFs in breast cancer were heterogenous with no trend in the expression [116,117]. This phenotypic heterogeneity of CAFs is not the only heterogeneity seen with CAFs. Lineage-dependent heterogeneity and functional/molecular heterogeneity are also seen. Lineage-dependent heterogeneity is attributed to the numerous potential sources of CAFs as previously mentioned. Interaction of CAFs with the tumor cells and other stromal cells also results in a change in molecular expression and function over time, resulting in spatial and temporal heterogeneity [115,118]. Amongst all these CAF surface receptors, the most relevant in oncology and nuclear medicine has been fibroblast activated protein (FAP) [113]. Kratochwil et al. are among the first researchers to image FAP expression with [^68^Ga]Ga-FAPi-04, a radiolabeled FAP inhibitor, in multiple cancers including breast cancer. Substantially high SUV values were seen in breast cancer [119]. Figure 3 shows a case of a patient imaged with [^68^Ga]Ga-FAPi-04 PET/CT. The complementary role of [^68^Ga]Ga-FAPi-04 PET/CT to the standard-of-care [^18^F]FDG PET/CT has been demonstrated in both preclinical and clinical settings [120,121]. In other scenarios, [^68^Ga]Ga-FAPi-04 PET/CT performed better than [^18^F]FDG PET/CT. This was especially true in brain, hepatic and peritoneal metastases [122,123,124]. The performance of [^68^Ga]Ga-FAPi-04 PET/CT among different breast cancer immunohistochemical subtypes remains to be evaluated. Figure 4 shows a patient with metastatic lobular breast cancer imaged with [^68^Ga]Ga-FAPi-04 in our center with a metastatic ‘superscan’ pattern. This could be important in cases where [^18^F]FDG PET/CT falters. An important study is that of Ballal et al. on the theranostic potential of [^68^Ga]Ga-DOTA.SA.FAPi PET/CT-guided [^177^Lu]Lu-DOTA.SA.FAPi radionuclide therapy [125].

#### 3.2.2. Neovasculature/Angiogenesis

##### Prostate-Specific Membrane Antigen (PSMA)

Although frequently expressed by prostate cancer and its metastases, expression of the PSMA has been demonstrated in neovascular capillary endothelium in the peripheral areas of a variety of epithelial malignancies [126,127]. Sathekge et al., demonstrates uptake of [^68^Ga]Ga-PSMA-11 in both female and male breast cancers [128,129,130]. Marafi et al., explain the uptake of [^18^F]F-PSMA-1007 in brain metastases from breast cancer [131]. This is an advantage over [^18^F]FDG PET which can potentially miss brain metastases due to its physiological brain uptake [131,132]. These results have provided an opportunity for PSMA-targeted radionuclide therapy.

##### Integrins Recognizing Arginine-Glycine-Aspartate (RGD)

Integrin αvβ3 with arginine-glycine-aspartate (RGD) recognize that cell surface integrin is upregulated on endothelial cells during angiogenesis seen during tumorigenesis and in tumor cells. The integrins play a role in tumor growth, invasiveness, metastases and angiogenesis [133]. Both SPECT and PET tracers such as [^99m^Tc]Tc-3PRGD2 and [^18^F]F-alfatide, respectively, have been evaluated, demonstrating potential in response monitoring and complementary roles to [^18^F]FDG PET/CT [134,135,136]. However, larger studies remain a necessity.

### 3.3. Other Imaging Targets (Cellular Proliferation, Hypoxia, Fatty Acid Synthesis and Somatostatin Receptor Imaging)

Cellular proliferation: Cancers are characterized by uncontrolled cellular growth and DNA replication. DNA replication can be imaged with radiolabeled analogous of pyrimidine and thymidine such as [^18^F]fluorothymidine ([^18^F]FLT) [137,138]. At the cellular level, the uptake of [^18^F]FLT demonstrates correlation with the prognostic and cellular DNA proliferation marker, the Ki-67 index, showing its potential as a surrogate marker of proliferation in primary breast cancer [139]. [^18^F]FLT has also shown potential in early response assessment in breast cancer patients receiving DNA-targeting chemotherapies [138,140].

Hypoxia imaging: As the tumor proliferates uncontrollably, it may outgrow its own blood supply, rendering it hypoxic [141]. This hypoxia has been imaged with tracers such as [^18^F]flouromisonidazole([^18^F]FMISO) and [^68^Ga]Ga-Tri-imidazole [142,143]. [^18^F]FMISO has shown a strong correlation to adverse outcomes compared to [^18^F]FDG. It has also shown potential in uncovering dynamic changes seen in the TME during taxane-based chemotherapy in TNBC; noting a decrease in uptake in responders and predicting resistance to endocrine therapies [144,145,146].

Fatty acid synthesis and somatostatin receptor targeting: Other tracers with the potential for breast cancer imaging include [^18^F]fluciclovine, somatostatin receptor imaging agents that image fatty acid synthesis and somatostatin receptor expression, among others [147,148]. These however require further exploration comparing them to the current standard-of-care.

### 3.4. Poly (ADP-Ribosyl) Polymerase (PARP) Imaging

PARP enzymatic activity is upregulated as part of the DNA damage response to single-strand DNA damage. Amongst the 18 PARP isoenzymes, PARP-1 accounts for 99% of the enzymatic activity [149]. These enzymes catalyze the conversion of nicotinamide adenine dinucleotide (NAD+) to polymers of poly (ADP-ribosyl) polymerase (PAR), which results in the recruitment of other DNA repair machineries [150]. Although this may be desirable under normal physiological conditions, in oncology it confers resistance to DNA-damaging therapies. This makes PARP inhibitor therapy such as rucaparib, olaparib, talozaparib and nucaparib feasible options in breast cancers with upregulated PARP. These PARP inhibitors have demonstrated significant benefits in tumors harboring homologous repair deficiencies such as *BRCA1/2* germline mutations, which are particularly common with TNBC [17,151,152,153]. The improved survival outcomes are as a result of ‘*synthetic lethality*’ due to the inability to repair single-strand DNA breaks and double-strand DNA breaks. These are also caused by PARP inhibitor therapy and homologous recombination deficiencies [154,155]. ‘BRCAness‘ is not limited to TNBC as homologous recombination deficiencies are seen in 8–10% of ER-positive breast cancer and are more commonly seen in young patients (<40 years). The majority of these are *BRCA1/2* mutations. PARP inhibitor therapy has demonstrated effectiveness in these hereditary ER-positive breast cancers [13].

Proper selection of patients likely to benefit from PARP inhibitor therapies is important as PARP inhibitors are expensive [156]. *BRCA1*/2 and other biomarkers testing have been used however with limited benefits seen [157,158]. A similar situation is seen with immunohistochemistry which can assess PARP upregulation in a single site without considering other sites of disease. Furthermore, primary and secondary resistance mechanisms may emerge during therapies, making non-invasive whole-body imaging an indispensable tool for imaging PARP upregulation [146]. Various imaging tracers based on the scaffolding of the respective PARP inhibitors have been investigated and some are undergoing clinical trials (see Table 2) [159,160].

[^18^F]Fluorothanatrace([^18^F]FTT) is one of the tracers that has reached clinical trials (NCT03083288, NCT03846167, NCT05226663 and NCT03604315) [161]. It has shown potential as a PET-based biomarker for imaging PARP-1 expression in breast cancer and ovarian cancer. It has also demonstrated potential for measuring pharmacokinetics of PARP inhibitors based on blocking studies conducted both clinically and pre-clinically. MacDonald et al. reported variability in vivo PARP expression based on imaging with [18]FTT independent of traditional predictors of increased expression. This included both non-carriers and carriers of *BRCA* pathogenic variants [162]. Other reports that did not necessarily image breast cancer did not portray additional aspects that may be clinically relevant in the future. These include the following:

- Biomarker of response to PARP inhibition adjuvant to radiotherapy [163].
- Early monitoring of therapeutic efficacy of PARP inhibitor therapy [164].
- Distinguishing between treatment-related/inflammation changes and residual/recurrent disease [165,166,167].
- More accurate delineation of tumor compares to the standard-of-care [^18^F]FDG PET/CT for radiotherapy planning [168,169].
- Theranostics: Selection of patients for radiolabeled PARP inhibitor therapy such as with Auger-emitting [^123^I]I/[^125^I]I and alpha-targeted therapies that have demonstrated preclinical efficacy in a variety of tumors [170,171,172,173,174,175,176].

Most studies have used SUV values as representative of PARP upregulation on PET-based PARP imaging. Whole-body PARP expression, which may be derived similarly as the semi-quantitative parameters for [^18^F]FDG, is likely to complement SUV values. The utility of these respective counterparts, total PARP-tumor volume (Total P-TV) and whole-body total lesion PARP expression (WTL-PE), remain to be evaluated. Comparison of uptake pattern to the standard-of-care [^18^F]FDG PET/CT in patients with breast cancer- based on the immunohistochemical subtype and *BRCA* germline pathogenic variant is yet to be determined.

## 4. Conclusions and Future Directions

**Figure 5:**
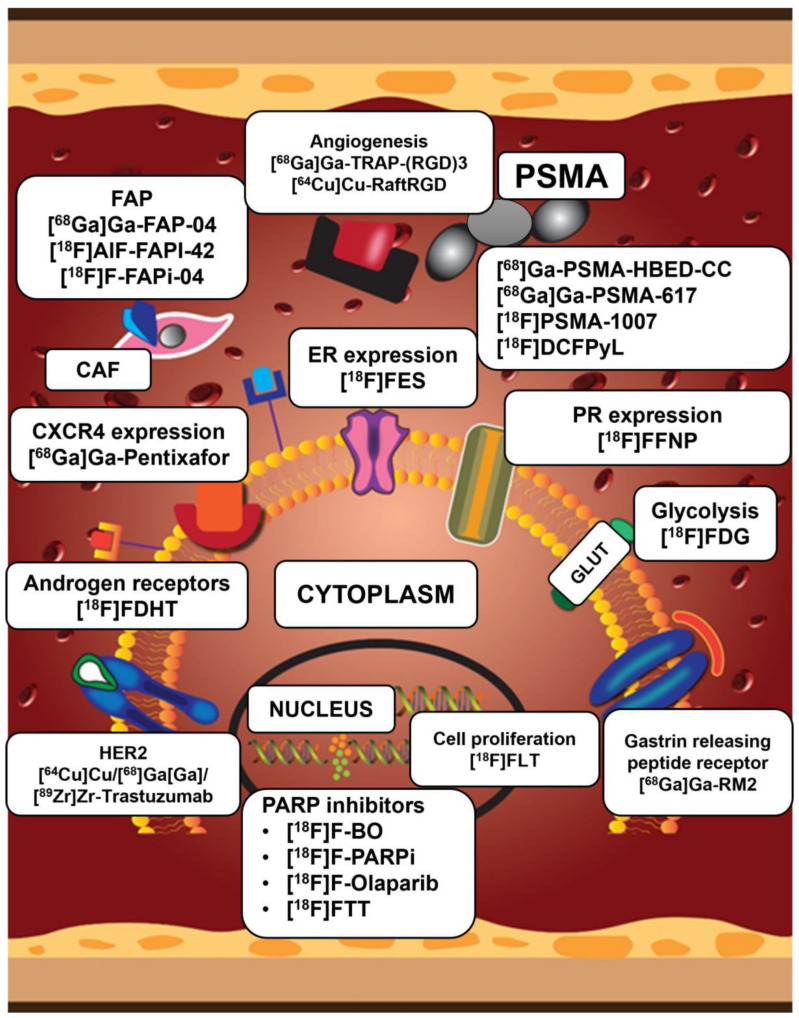
This depicts the various targets of the different PET imaging agents in breast cancer. These range from targets within the nucleus, cytoplasm, cell membrane and the microtumor environment. Within the microtumor environment, these target the cancer-associated fibroblasts’(CAF) fibroblast activated protein (FAP) and the microvasculature. PSMA: prostate specific membrane antigen; CAF: cancer-associated fibroblasts; FAPi: fibroblast activated protein inhibitor; HER2: human epidermal growth factor receptor-2; FES: 16-[^18^F]-fluoro-17-estradiol; CXCR4: C-X-C chemokine receptor type 4; FDHT: 16β-fluoro-5α-dihydrotestosterone; FFNP: fluorofuranylnorprogesterone; FDG: 2-deoxy-2-fluoro-D-glucose, GLUT: glucose transporter; FLT: fluorothymidine; RM2: bombesin.

Individualized breast cancer care comprises taking clinicopathological and immunohistochemical characteristics into account. Immunohistochemistry has drawbacks that may be overcome by whole-body in vivo imaging methods. Although conventional imaging has typically been employed in the staging and decision-making of breast cancer imaging, molecular imaging, which images specific molecular targets and metabolic pathways, has gained popularity. These technologies enable prompt decision-making, which eventually improves outcomes in breast cancer patients. The widely accessible standard-of-care metabolic imaging probe [^18^F]FDG PET/CT, which has been validated in staging, response evaluation, recurrence assessment and prognostication, is non-specific. Furthermore, despite its great impact, it falls short in some histological and IHC breast cancer subtypes. This has led to the development of imaging modalities targeting receptor(s) expression, neo-vasculature, tumor microenvironment components and the DNA damage repair processes. A typical example is [^18^F]FES PET/CT which has been approved by the FDA for various indications in the evaluation of ER-positive breast cancer. Figure 5 shows a summary of both approved and investigation tracers in breast cancer imaging targeting various molecular pathways and receptors in breast cancer.

The recent approval of PARPi treatment in breast cancers with homologous DNA recombination defects and temporal or geographic heterogeneity in PARP overexpression/upregulation highlights the necessity for a non-invasive whole-body in vivo imaging approach which can be used in conjunction with IHC. These have been developed and have been shown to be feasible and safe. Their prospective applications in breast cancer include PARPi therapy decision-making, staging, monitoring PARP upregulation in response to conventional therapies, prognosis, and radionuclide treatment guidance. However, more research is needed to define the uptake pattern in different breast cancer categories, as well as the criteria for semi-quantitative and visual analysis of the respective PET/CT images, including comparison with the standard-of-care [^18^F] FDG PET/CT.

## Figures and Tables

**Table 1:** Semi-quantification of lesional and whole-body [^18^F] FDG tumor uptake on PET/CT.

Parameter	Definition
Standardized uptake value (SUV)	The ratio of the image-derived radioactivity concentration and the whole-body concentration of the injected activity.
Mean standardized uptake value (SUVmean)	Calculated by dividing mean tissue activity in the region or volume of interest by the injected activity normalized to patient body weight.
Maximum standard uptake value (SUVmax)	The maximum tissue activity within the region or volume of interest is divided by the injected activity normalized to the patient’s body weight.
Peak standardized uptake value (SUVpeak)	This is calculated as the average of the SUV within a small, fixed region of interest (ROI) centered on a high-activity part of the lesion.
Lean standardized uptake value (SUL)	The tissue activity divided by the lean body mass of the patient.
Mean tumor volume (MTV)	The volume of the lesion with non-physiological uptake of [^18^F]FDG. This will be summed up to obtain the total metabolic tumor volume.
Total lesion glycolysis (TLG)	SUVmean of a single lesion multiplied by its respective MTV. These will be summed up for all the lesions to obtain the TLG.

**Table 2:** Summary of tracer targeting PARP upregulation based on PARP inhibitor scaffolding.

	PET/SPECT Tracer	Tracer
Olaparib-based molecular probes	PET	[^18^F]F-BO
		[^18^F]F-PARPi-FL
		[^18^F]F-PARPi
		[^18^F]F-Olaparib
		[^18^F]F-20
		[^18^F]F-9e and [^18^F]F-AZD2461
		[^18^F]FPyPARP
		[^68^Ga]Ga-DOTA-Olaparib
		[^11^C]C-Olaparib
		[^64^Cu]Cu-DOTA-PARPi
	SPECT	[^123^I]I-PARPi
		[^131^I]I-PARPi
		[^131^I]I_2_-PARPi
		[^123^I]I-MAPi
		[^125^I]I-PARPi-01
Rucaparib-based molecular probes	PET	[^18^F]FTT
		[^18^F]F-WC-DZ-F
		[^18^F]F-rucaparib
	SPECT	[^123^/^125^I]I-KX1
Talazoparib-based molecular probes	PET	[^18^F]F-talazoparib
Molecular probes based on other PARP inhibitors	PET	[^18^F]F-SuPAR
	SPECT	[^125^I]I-KX-02-019

## Data Availability

The articles quoted and referenced are available online as referenced. The images used for the review are available from the corresponding author on request.
